# *Kandelia candel* Thioredoxin f Confers Osmotic Stress Tolerance in Transgenic Tobacco

**DOI:** 10.3390/ijms21093335

**Published:** 2020-05-08

**Authors:** Xiaoshu Jing, Jun Yao, Xujun Ma, Yanli Zhang, Yuanling Sun, Min Xiang, Peichen Hou, Niya Li, Rui Zhao, Jinke Li, Xiaoyang Zhou, Shaoliang Chen

**Affiliations:** 1 Beijing Advanced Innovation Center for Tree Breeding by Molecular Design, College of Biological Sciences and Technology, Beijing Forestry University, Beijing 100083, China; johncy@126.com (X.J.); yaojun990@126.com (J.Y.); z585788@163.com (Y.Z.); 18003251998@163.com (Y.S.); ruizhao926@126.com (R.Z.); jinkeli@bjfu.edu.cn (J.L.); zhouxiaoyang@bjfu.edu.cn (X.Z.); 2 Shandong University-Helmholtz Institute of Biotechnology, State Key Laboratory of Microbial Technology, School of Life Science, Shandong University, Qingdao 266237, China; 3 Urat Desert-Grassland Research Station, Northwest Institute of Eco-Environment and Resources, Chinese Academy of Science, Lanzhou 730000, China; maxujun@lzb.ac.cn; 4 Department of Biology, College of Life Science, Hainan Normal University, Haikou 571158, China; x8113462@163.com (M.X.); niyali6515@163.com (N.L.); 5 Beijing Research Center of Intelligent Equipment for Agriculture, Beijing Academy of Agriculture and Forestry Sciences, Beijing 100097, China; houpc@nercita.org.cn

**Keywords:** thioredoxin, *Kandelia candel*, mannitol, drought, water retaining capacity, soluble sugar, H_2_O_2_, stomatal aperture, abscisic acid, K^+^ flux, guard cells, noninvasive micro-test technique

## Abstract

Water deficit caused by osmotic stress and drought limits crop yield and tree growth worldwide. Screening and identifying candidate genes from stress-resistant species are a genetic engineering strategy to increase drought resistance. In this study, an increased concentration of mannitol resulted in elevated expression of thioredoxin f (*KcTrxf*) in the nonsecretor mangrove species *Kandelia candel*. By means of amino acid sequence and phylogenetic analysis, the mangrove Trx was classified as an f-type thioredoxin. Subcellular localization showed that KcTrxf localizes to chloroplasts. Enzymatic activity characterization revealed that KcTrxf recombinant protein possesses the disulfide reductase function. *KcTrxf* overexpression contributes to osmotic and drought tolerance in tobacco in terms of fresh weight, root length, malondialdehyde (MDA) content, and hydrogen peroxide (H_2_O_2_) production. KcTrxf was shown to reduce the stomatal aperture by enhancing K^+^ efflux in guard cells, which increased the water-retaining capacity in leaves under drought conditions. Notably, the abscisic acid (ABA) sensitivity was increased in *KcTrxf*-transgenic tobacco, which benefits plants exposed to drought by reducing water loss by promoting stomatal closure. *KcTrxf*-transgenic plants limited drought-induced H_2_O_2_ in leaves, which could reduce lipid peroxidation and retain the membrane integrity. Additionally, glutathione (GSH) contributing to reactive oxygen species (ROS) scavenging and transgenic plants are more efficient at regenerating GSH from oxidized glutathione (GSSG) under conditions of drought stress. Notably, *KcTrxf*-transgenic plants had increased glucose and fructose contents under drought stress conditions, presumably resulting from KcTrxf-promoted starch degradation under water stress. We conclude that KcTrxf contributes to drought tolerance by increasing the water status, by enhancing osmotic adjustment, and by maintaining ROS homeostasis in transgene plants.

## 1. Introduction 

Drought is a major natural disaster that not only reduces the crop yield but also limits the growth and productivity of forest trees [1,2]. Global warming has resulted in more frequent and extreme occurrences of drought [3,4]. Genetic engineering is considered a functional tool that enhances drought resistance in crop and tree species [2]. Screening and identifying candidate genes from stress-resistant species are a strategy for drought tolerance engineering. True mangroves along coastlines are interesting models for screening candidate genes since they are able to tolerate the extreme osmotic stress caused by the seawater level of salt [5,6,7,8]. *Kandelia candel* is a major mangrove species that occurs along southern China coastlines. NaCl-altered photosynthesis, salt accumulation, ion compartmentation, and fluxes have previously been investigated in nonsecretor mangroves [9,10,11]. *K. candel* plants have an oxygen-scavenging system that acts against reactive oxygen species (ROS) under conditions of NaCl stress in addition to the Na^+^/H^+^ antiport system to remain in ionic homeostasis [12,13,14]. *K. candel* increases the transcription of the *CSD* gene which encodes a Cu/Zn superoxide dismutase (SOD) to reduce ROS in chloroplasts over the long term and in a high saline environment [12]. Moreover, salt treatment increases the transcription of an f-type thioredoxin (Trx) in *K. candel* [13]. Being small and ubiquitous proteins (12–14 kD) with a conserved redox active site (WCXPC), Trxs serve as a crucial important redox regulator in higher plants [15]. These proteins are able to catalyze the reduction of disulfide bonds in many target proteins to regulate their structure and function [15]. We showed that *KcTrxf*-transgenic plants can scavenge the salt-elicited ROS in leaf cells through the upregulation of catalase and ascorbate peroxidase (APX) and can increase the activity of monodehydroascorbate reductase (MDAR) and glutathione reductase (GR) in the chloroplast ascorbate–glutathione (AsA–GSH) cycle [16,17,18], leading to an increase in reduced glutathione (GSH) and nonprotein thiols (NPTs) in the leaves [13]. Antioxidative systems also play an important role in scavenging ROS and in controlling the cellular redox potential against oxidative stress [19,20,21]. However, the regulatory roles of *K. candel* Trx family genes in osmotic and drought tolerance are not yet fully understood.

The plant water status is tightly controlled by stomatal control on transpiration and osmotic adjustment [22,23,24]. Stomatal regulation is one of the most important measures to reduce water loss when soil water availability to plants is limited [25,26]. Endogenous abscisic acid (ABA) is rapidly produced upon water deficit, osmotic, and salt stress [27,28,29,30,31], initiating a signaling cascade that results in stomatal closure [32,33,34,35,36]. Stomatal closure requires potassium (K^+^) to exit from the guard cells [34]. The outward-rectifying K^+^ channels mediate the K^+^ flow in the plasma membrane (PM) of guard cells [34,35]. ABA results in depolarization of PM, which deactivates inward-rectifying K^+^ channels but stimulates outward-rectifying K^+^ channels, resulting in K^+^ efflux from guard cells [34,35]. The K^+^ efflux from guard cells contributes to a loss of guard cell turgor and to stomatal closing [36]. Histochemical localization of β-glucuronidase (GUS) expression at the *PsTRXf1*:GUS promoter revealed that pea chloroplastic Trxf is expressed in guard cells [37]. However, the roles of *K. candel* Trxf in the regulation of the stomatal aperture and ABA response remain to be clarified.

Mangroves possess more potential ability to carry out osmotic adjustment to maintain cell turgor under osmotic stress [5,6]. *K. candel* tends to accumulate Na^+^ within the cytoplasm, vacuoles, and chloroplasts in leaves [9,14]. This helps the mangrove to maintain a positive turgor pressure under osmotic conditions [5,6,38]. Moderate drought results in increased concentrations of soluble carbohydrates and polyols, which potentially promotes the maintenance of cell turgor in *Populus euphratica* leaves through increased osmotic pressure [39]. It is noteworthy that the concentrations of amino acids, in particular, proline, increase drastically in both water- and salt-stressed poplars [40,41,42,43,44]. Type-f thioredoxins have various functions in the mediation of carbon metabolism [15,45,46,47,48,49]. Overexpression of *NtTrx f* increases starch accumulation in tobacco leaves [45], and knockout of *AtTrx f1* decreases the starch content in the leaves of *Arabidopsis thaliana* mutants [46]. In contrast, thioredoxin f has been proposed to participate in a redox-regulated pathway of starch degradation under specific stress conditions [47,48,49]. For example, thioredoxin-regulated β-amylase (BAM1) triggers diurnal starch degradation in guard and mesophyll cells under conditions of osmotic stress [48]. Little is known about the role of *K. candel* Trxf in the regulation of carbon metabolism in response to osmotic stress.

The objective of this study was to explore the role of *KcTrxf* in plants adapting to water-limited environments. In this study, the *Trxf* gene was cloned from the nonsecretor mangrove species *Kandelia candel*. Subcellular localization analysis revealed that KcTrxf localized to the chloroplasts. The promoter activity of *KcTrxf* was investigated by GUS reporter gene expression in transgenic Arabidopsis. Trx activity was analyzed using the purified recombinant KcTrxf protein. *KcTrxf* was transferred to *Nicotiana tabacum* to clarify its role in the modulation of osmotic and drought tolerance. The leaf water-retaining capacity; stomatal aperture; malondialdehyde (MDA) content; H_2_O_2_ production; GSH and oxidized glutathione (GSSG) contents; and contents of soluble sugars, such as sucrose, glucose, and fructose, were examined under conditions of osmotic or drought stress. Furthermore, the stomatal sensitivity to ABA and K^+^ flux in the PM of guard cells was compared between wild-type and *KcTrxf*-overexpressing plants. Our data showed that *KcTrxf* contributes to the maintenance of water status by increasing stomatal sensitivity and by enhancing osmotic adjustment, which helps the transgene plants to limit H_2_O_2_ production under drought conditions. The increased ratio of GSSG/GSH in *KcTrxf*-transgenic plants indicates that GSH contributes to ROS scavenging under conditions of drought stress. Consequently, the negative consequences of stress-induced ROS are alleviated in droughted plants.

## 2. Results

### 2.1. Expression Profile of KcTrxf Following Exposure to Mannitol in Kandelia Candel Leaves

**Figure 1:**
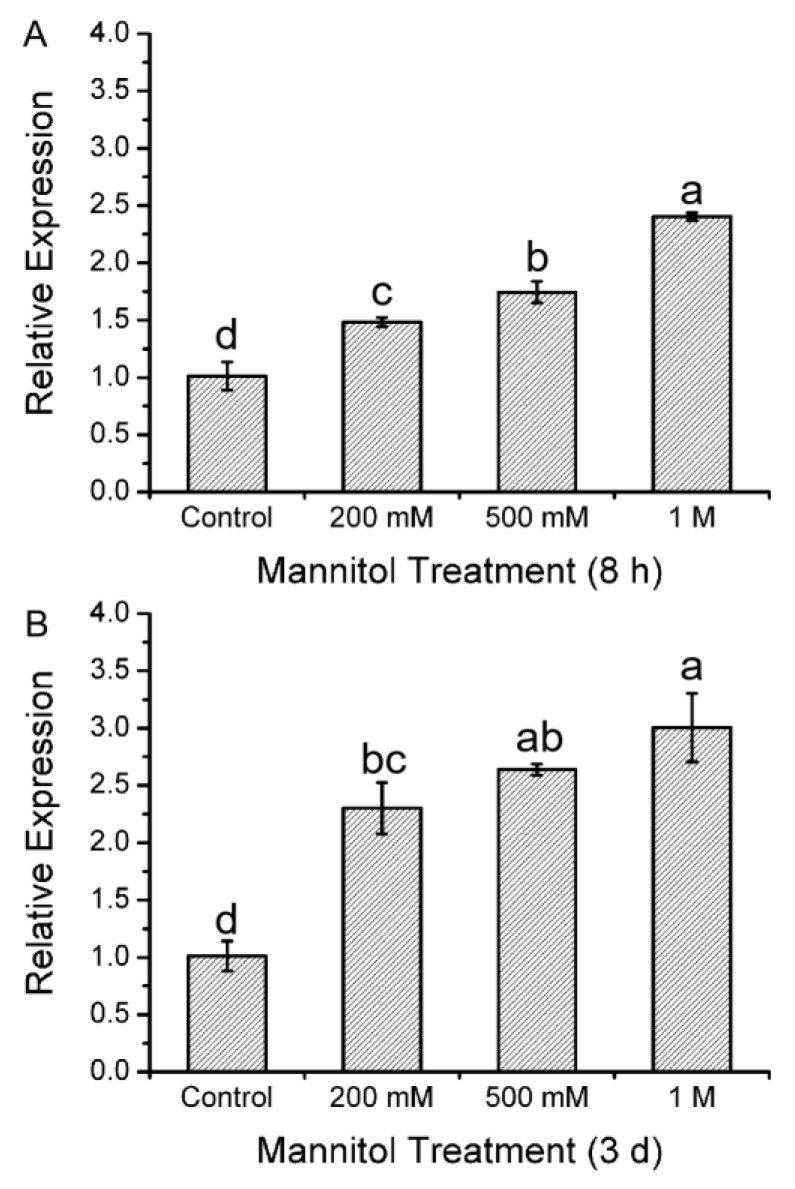
Expression level of the thioredoxin f (*KcTrxf*) gene in *Kandelia candel* leaves following mannitol treatment: *Kandelia candel* seedlings were exposed to 0, 200 mM, 500 mM, or 1.0 M of mannitol in Hoagland nutrient solution for 8 h (**A**) or 3 days (**B**). Then, the upper leaves were collected for real-time quantitative polymerase chain reaction (PCR) analysis. Expression levels of *KcTrxf* were normalized to the *Kandelia candel* housekeeping gene, *Tublin*, as an internal reference. Each column corresponds to the mean of three individual plants, and bars represent the standard error of the mean. Columns labeled with different letters, a, b, c, and d, represent significant differences at *p* < 0.05 between the wild-type (WT) and transgenic lines.

To determine the possible involvement of *KcTrxf* in response to osmotic stress, the expression pattern was analyzed by real-time quantitative PCR (Figure 1). The exposure to osmotic stress caused by mannitol at 200 mM, 500 mM, or 1.0 M resulted in elevated *KcTrxf* expression (Figure 1). The pattern of osmotic stress-induced *KcTrxf* after short-term (8 h, Figure 1A) treatment was similar to that following long-term exposure (3 days, Figure 1B). The expression of *KcTrxf* increased as the concentration of mannitol increased, reaching the highest level at 1.0 M (2.5–3.2-fold) (Figure 1). This result shows that *KcTrxf* expression is induced by osmotic stress in the mangrove species.

### 2.2. KcTrxf Cloning and Sequence Analysis

**Figure 2:**
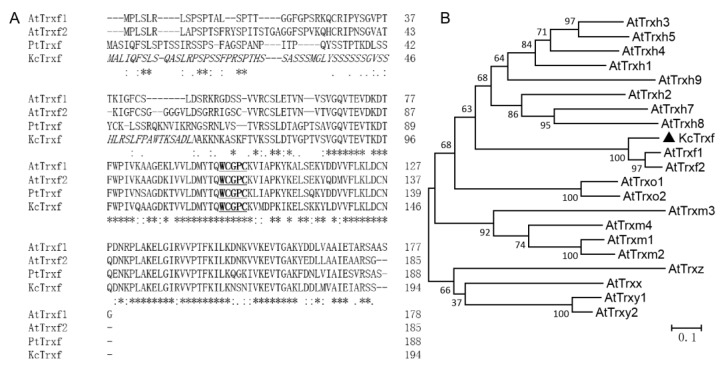
Amino acid sequence and phylogenetic analysis of KcTrxf: (**A**) Multiple sequence alignment of KcTrxf with Trxfs from other species. Amino acid sequences of Trxf were deduced from *Kandelia candel* (KcTrxf), *Populus trichocarpa* (PtTrxf), and *Arabidopsis thaliana* chloroplasts (AtTrxf1 and AtTrxf2). Asterisks (*) and dots (·, :) indicate identical and conserved amino acid residues, respectively. Italics represent chloroplast transit peptides. Active sites are underlined and in bold. (**B**) Phylogenetic relationships between KcTrxf and other Trx family members from different species. The phylogenetic tree was constructed with the neighbor-joining method using MEGA 5. Bootstrap values of 1000 replicates are presented.

The 585-bp full-length cDNA of *KcTrxf* was cloned from *K. candel* leaves. The cDNA sequence encoded a putative protein of 194 amino acids (Figure 2A). The protein contains the canonical active site-WCGPC motif. Multiple sequence alignment revealed that KcTrxf displays high identity with the Trxfs in other plant species (Figure 2A). KcTrxf showed that 55.84% identifies with AtTrxf1 and that 56.35% identifies with AtTrxf2. The constructed phylogenetic tree showed the conservation of KcTrxf to other Trxfs (Figure 2B). Therefore, KcTrxf was classified as an f-type thioredoxin.

### 2.3. Subcellular Localization of KcTrxf

**Figure 3:**
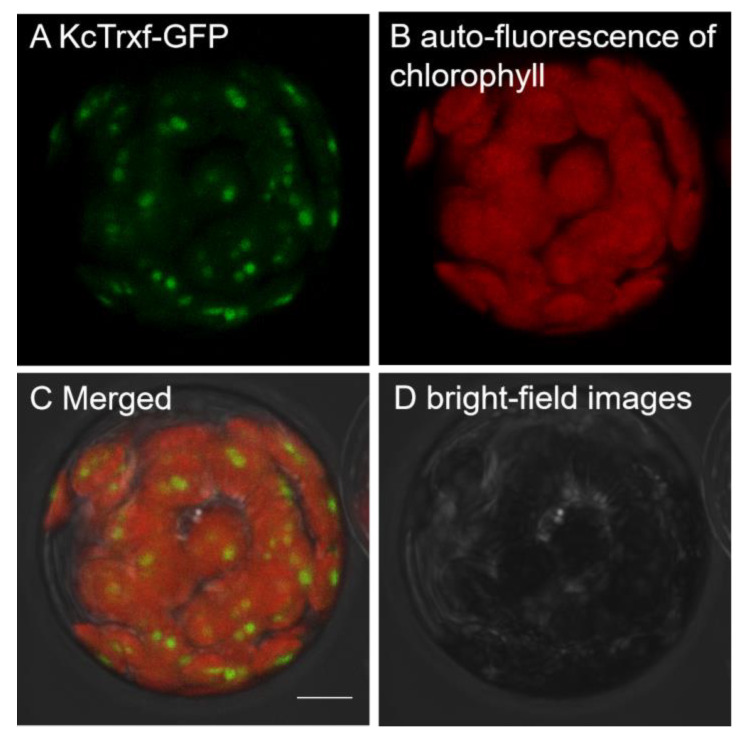
Subcellular localization of KcTrxf in Arabidopsis mesophyll protoplasts: Representative images of green fluorescence of KcTrxf-GFP (**A**) and red autofluorescence of chlorophyll (**B**) were monitored separately using a confocal laser scanning microscope and the two colored fluorescence images were merged (**C**). (**D**) Bright-field images. Bar = 10 μm.

The subcellular location predicted by TargetP [50] indicated that *K. candel* Trxf might be localized to chloroplasts, and ChloroP [51] predicted that a chloroplast transit peptide of 62 amino acid is cleaved (Figure 2A). To confirm the localization of *K. candel* Trxf, a construct containing a translational fusion of its open reading frame to the green fluorescent protein (GFP) reporter gene at its C terminus was transiently expressed in *Arabidopsis* protoplasts and imaged using confocal laser microscopy. The fluorescence emitted by the GFP reporter of the fusion construct was targeted to the chloroplast, showing punctate structures in the chloroplast (Figure 3). The KcTrxf location is consistent with our previous report in a salt study, where KcTrxf was shown to be a typical thioredoxin in the chloroplast [13]. The vector control pEZS-NL did not express GFP without adding a coding sequence to the 5′ end of the open reading frame [13].

### 2.4. KcTrxf Promoter:GUS Fusion in Transgenic Arabidopsis Plants

**Figure 4:**
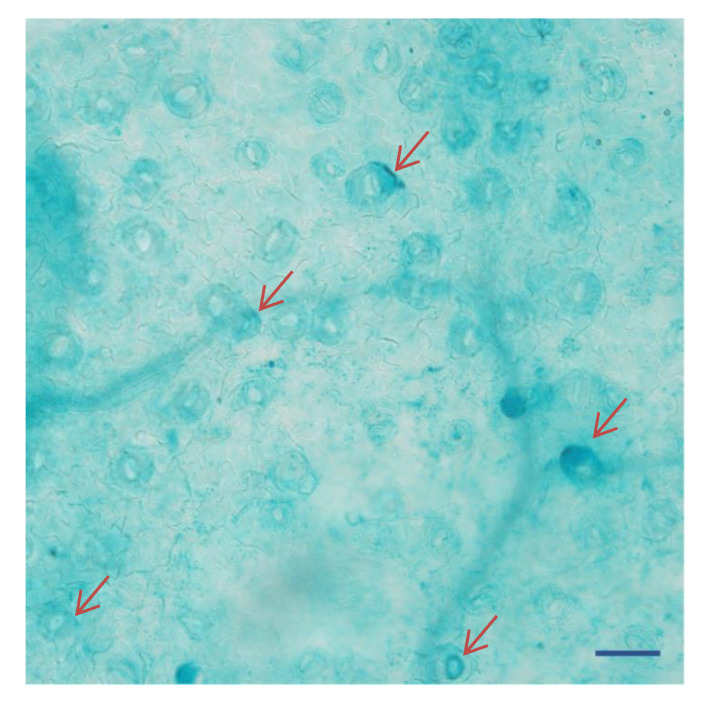
Histochemical localization of β-glucuronidase (GUS) expression in guard cells under the control of the *KcTrxf* promoter in transgenic *Arabidopsis* plants: The representative figure shows a typical expression pattern obtained from two independent experiments. The arrows indicate the dense staining of several guard cells. Bar = 100 μm.

To examine the contribution of the 5′ region of *KcTrxf* genes to the regulation of expression, extensions of 5′ flanking sequences of *KcTrxf* fused to the GUS reporter gene were expressed in *Arabidopsis.* Specific expression in guard cells was confirmed in GUS plants (Figure 4). Our data is in agreement with that obtained by de Dios Barajas-Lopez et al. (2007) [37], who found that the promoter of pea thioredoxin f is active in guard cells of transgenic Arabidopsis. Furthermore, GUS activity was observed in mesophyll cells in addition to in guard cells (Figure 4). The staining of mesophyll cells reduced the appearance of GUS in guard cells (Figure 4). Similarly, strong GUS expression was also seen in the leaves of *PsTRXf1*::GUS promoter transgenic Arabidopsis plants [37].

### 2.5. Purification of Recombinant KcTrxf Protein and Trx Activity

**Figure 5:**
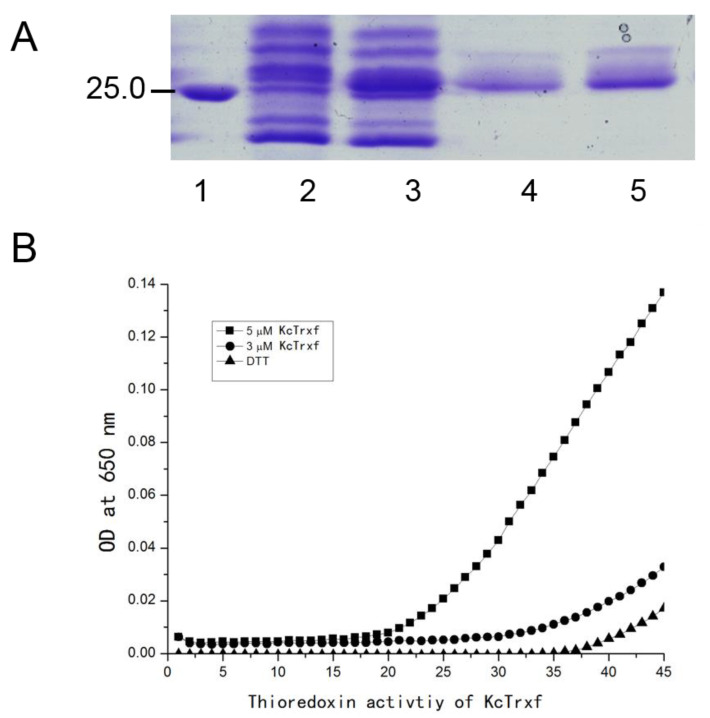
Purification of KcTrxf recombinant protein and thioredoxin activity assay. (**A**) SDS-PAGE of KcTrxf recombinant protein: Crude extract of *Escherichia coli* transformed with Pet28a-*KcTrxf* before (Lane 2) and after IPTG induction (Lane 3). KcTrxf was purified by sephadex gel filtration (Lanes 4 and 5). Molecular weight markers (Lane 1) are shown in kilodaltons. (**B**) The activity of the KcTrxf protein determined by insulin disulfide reduction assay: The reduction of insulin was measured by an increase in turbidity at 650 nm because of insulin precipitation. The reaction containing 3 μM KcTrxf (●) and 5 μM KcTrxf (■) began by dithiothreitol (DTT) addition. The nonenzymatic insulin reduction by DTT alone (▲) was used as a negative control.

It has been shown that the peculiar character of Trx proteins has a disulfide reductase function [52,53,54]. Using dithiothreitol (DTT) as the reductant, the intermolecular disulfide bonds between the insulin A and B chains can be reduced by Trx. Precipitation of the insoluble B chain can be measured photometrically by an increase in the absorbance at 650 nm [55]. Therefore, to determine whether *K. candel* Trxf possesses disulfide reductase activity, the proteins were expressed in *E. coli* to obtain recombinant proteins. The recombinant proteins were purified by sephadex gel filtration and analyzed with sodium dodecyl sulfate-polyacrylamide gel electrophoresis (SDS-PAGE). KcTrxf accumulated at a high level in the soluble fraction after isopropylthythio-β-galactoside (IPTG) induction and was purified to homogeneity (Figure 5A).

Then, the activity of the recombinant protein was determined by reducing insulin-disulfide bridges in the presence of dithiothreitol (DTT) [55]. Compared with the negative control (DTT alone), the addition of recombinant protein resulted in an abrupt increase in turbidity at 650 nm (Figure 5B), indicating a reduction of insulin, as described previously [55]. In the reaction mixture, Trx activity increased with the concentration of KcTrxf recombinant protein from 3 or 5 μM (Figure 5B).

### 2.6. Overexpression of KcTrxf in Tobacco and Phenotype Tests

**Figure 6:**
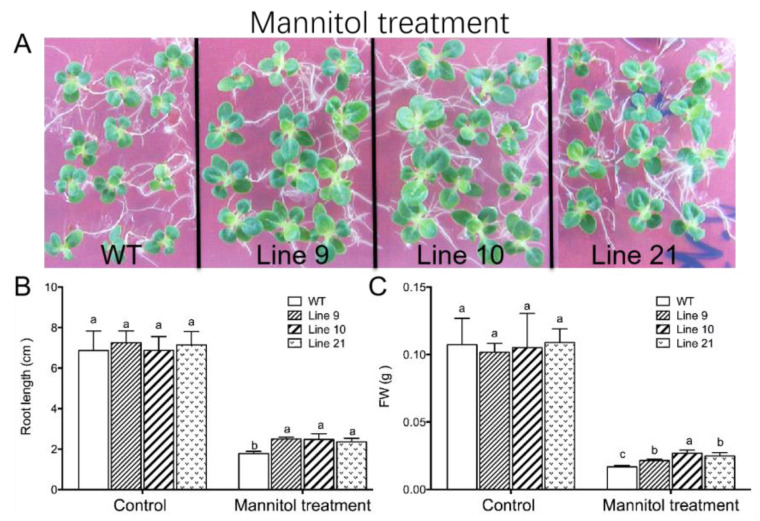
Phenotype tests of wild-type tobacco and *KcTrxf*-transgenic seedlings under osmotic treatment: Seeds of wild-type (WT) and transgenic lines (L9, L10, and L21; T2 generation) were germinated on MS medium for 7 days and then subjected to 0- or 250-mM mannitol for 14 days. Then, the root lengths and fresh weights of whole seedlings from WT and transgenic plants were measured. (**A**) Representative images showing plant performance after exposure to mannitol for 14 days. (**B**,**C**) The root growth and fresh weight of tobacco seedlings: In Figure 6B,C, each column is the mean of 12–20 individual plants and bars represent the standard error of the mean. Columns labeled with different letters, a, b, and c, represent significant differences between WT and transgenic lines at *p* < 0.05.

**Figure 7:**
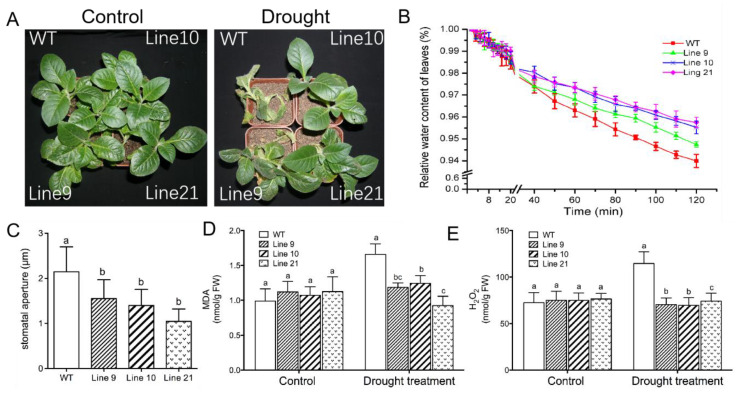
Phenotype tests of wild-type tobacco and *KcTrxf*-transgenic seedlings under conditions of drought stress: Four-week-old rooted plants on MS of WT and transgenic lines (L9, L10, and L21) were transferred to nursery soil for 4 weeks of acclimation and then exposed to drought treatment by withholding water for 2 weeks. (**A**) Representative images showing plant performance after exposure to drought stress for 14 days. (**B**) Water-retaining capacity (WRC): Fully opened leaves were sampled from WT and transgenic plants, and the WRC of the detached leaves was measured after exposure to air for 120 min. Each value is the mean of four individual plants, and bars represent the standard error of the mean. (**C**) Stomatal aperture: Epidermal peels were stripped from air-exposed leaves and used for stomatal aperture measurement. Each column is the mean of 100 stomata from four individual plants, and the bars represent the standard error of the mean. (**D**) Malondialdehyde (MDA) content. (**E**) H_2_O_2_ production. After 14 days of drought treatment, plants were harvested from WT and transgenic plants to measure MDA and H_2_O_2_ production. In Figure 7C–E, columns labeled with different letters, a, b, and c, represent significant differences between WT and transgenic lines at *p* < 0.05.

To testify the importance of *KcTrxf* under conditions of osmotic and drought tolerance, *KcTrxf* was transferred to tobacco under the control of the *CaMV* 35S promoter. Phenotypic screening was carried out using seedlings grown on MS medium and rooted plants acclimated to nursery soil. In brief, the following steps were carried out:

(i) Seven-day-old seedlings grown on MS medium were transferred to MS medium supplemented with 250 mM mannitol. After 14 days of mannitol treatment, the wild-type (WT) plants exhibited osmotic injury, e.g., smaller dark green leaves with slight curled edges (Figure 6A). No obvious injury symptom was observed in *KcTrxf*-transgenic plants (Figure 6A). The transgenic plants displayed higher root lengths and fresh weights than WT under mannitol treatment (Figure 6B,C). There were no significant differences between WT and transgenic plants in terms of root length and fresh weight under normal growth conditions (Figure 6B,C).

(ii) In a drought test, four-week-old seedlings were transferred and acclimated to nursery soil for four weeks. All genotypes were subjected to water stress by withholding water for two weeks. Following drought treatment, the wild-type plants wilted whereas *KcTrxf*-transgenic plants showed no sign of wilting (Figure 7A). Collectively, the phenotype tests showed that KcTrxf enhanced plant tolerance to osmotic stress and drought stress.

To determine whether *KcTrxf* alleviated drought-induced oxidative damage, malondialdehyde (MDA), a marker of lipid peroxidation, was measured. The MDA content increased significantly in the wild-type plants during drought treatment, while MDA remained at low levels in transgenic plants (Figure 7D). This indicates that the membrane integrity of transgenic plants was less disrupted by lipid peroxidation.

H_2_O_2_ levels were measured, as ROS might contribute to drought-induced lipid peroxidation in WT plants. H_2_O_2_ levels induced by drought were significantly higher in WT plants than in transgenic plants (Figure 7E), suggesting that KcTrxf was able to regulate ROS homeostasis and to reduce the oxidative damage caused by drought stress.

Being an important antioxidant in the AsA–GSH cycle, glutathione plays important roles in antioxidative defense in stressed plants [17,18,19]. Drought treatment decreased the GSH content in WT but increased the oxidized glutathione (GSSG) content, which led to an increase in the GSSG/GSH ratio (49%, Table 1). GSH was less affected by drought in *KcTrxf*-transgenic lines, although a typically low GSH content was observed under control conditions (Table 1). Drought induced an increase in the GSSG/GSH ratio by 9–35% due to the increased GSSG content in the transgenic lines of stressed plants.

### 2.7. KcTrxf Overexpression Increases Water-Retaining Capacity and Stomatal Sensitivity to ABA

Maintaining the water status is crucial for plants to adapt to a drought environment. The water-retaining capacity (WRC), which reflects the plant’s ability to control water loss under drought, was examined in both WT tobacco and *KcTrxf*-transgenic lines. The transgenic plants exhibited typically higher WRC than the wild-type plants after 2 h of air exposure (Figure 7B). These results indicate that the transgenic plants can retain their water content in response to drought stress.

The lower water loss in transgenic lines was, at least in part, due to closure of the stomata [56]. During the period of air exposure, the stomatal apertures in the transgenic plants were 36%–44% lower than in wild-type seedlings (Figure 7C). The low stomatal apertures reduced water loss during the period of air exposure in transgene plants.

### 2.8. Drought-Induced K^+^ Fluxes in Guard Cells

**Figure 8:**
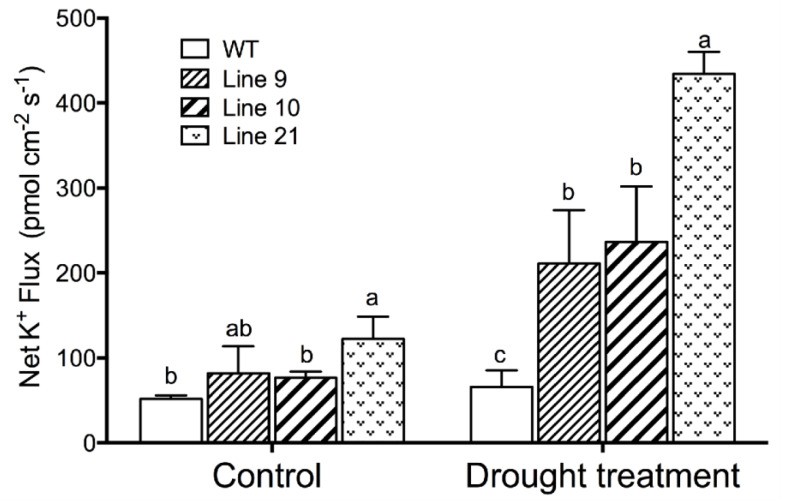
Steady K^+^ flux profiles in guard cells of WT and transgenic lines under drought treatment: Four-week-old rooted plants on the MS of WT and transgenic lines (L9, L10, and L21) were transferred to nursery soil for 4 weeks of acclimation and then exposed to drought treatment by withholding water for 2 weeks. The K^+^ fluxes in guard cells were measured using the noninvasive micro-test technique (NMT). Each column is the mean of 30–40 stomata from four individual plants, and bars represent the standard error of the mean. Columns labeled with different letters, a, b, and c, represent significant differences between WT and transgenic lines at *p* < 0.05.

Drought-induced stomatal closing is mediated by soluble osmolytes, for example K^+^, in the guard cells [36]. The K^+^ flux was measured in guard cells, since stomatal closure requires potassium to exit from the PM of guard cells [33]. Noninvasive micro-test technique (NMT) flux data revealed that guard cells of the transgenic plants exhibited a greater net K^+^ efflux than wild-type plants after drought stress (Figure 8).

### 2.9. KcTrxf Overexpression Increases Stomatal Sensitivity to ABA

**Figure 9:**
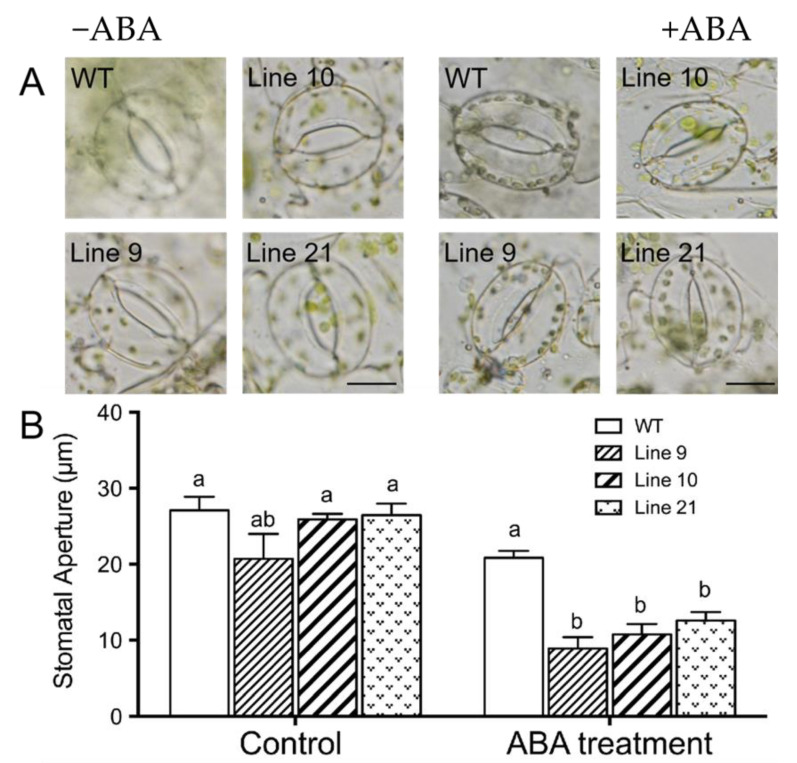
Abscisic acid (ABA)-induced stomatal closure in wild-type (WT) and *KcTrxf*-transgenic lines: Wild-type tobacco and *KcTrxf*-transgenic seedlings were subjected to 0 or 5 µM ABA treatment for 2 h after leaves were illuminated with cool light (150 μmol m^−2^ s^−1^) for 2 h to induce stomatal opening. Thereafter, epidermal peels were stripped from fully opened leaves of WT and *KcTrxf*-transgenic Arabidopsis lines (L9, L10, and L21). Stomatal apertures were measured in epidermal peels treated with or without ABA. (**A**) Representative images showing stomata before and after ABA treatment. Scale bar: 50 μm. (**B**) Stomatal aperture: Each column is the mean of 100 stomata from four individual plants, and bars represent the standard error of the mean. Columns labeled with different letters, a, and b, represent significant differences at *p* < 0.05 between WT and transgenic lines.

The phytohormone abscisic acid (ABA) induces stomatal closure, which is crucial for plant adaptation to water stress conditions [2]. To determine whether KcTrxf is involved in the ABA signaling pathway, the sensitivity to ABA was examined in *KcTrxf*-transgenic plants. At the tested dose (5 µM), abscisic acid increased stomatal closure in all tested lines, but a more pronounced enhancement was observed in transgenic lines (Figure 9A,B).

### 2.10. KcTrxf Increases Glucose and Fructose but Decreased Sucrose under Drought

**Figure 10:**
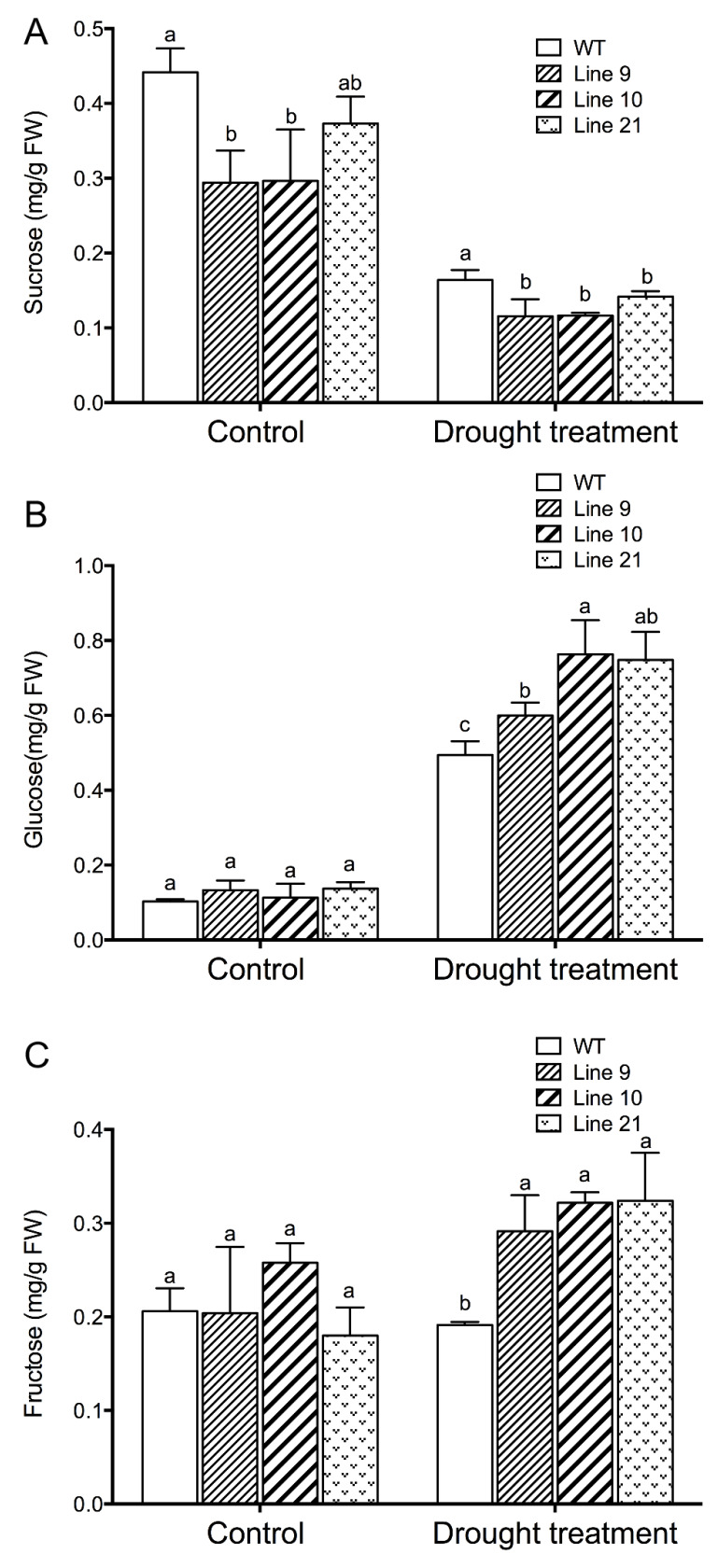
Sucrose, glucose, and fructose concentrations in the leaves of WT and transgenic lines under drought treatment: Four-week-old rooted plants on the MS of WT and transgenic lines (L9, L10, and L21) were transferred to nursery soil for 4 weeks of acclimation and then exposed to drought treatment by withholding water for 2 weeks. Leaves were sampled from control and water-stressed plants for sucrose (**A**), glucose (**B**), and fructose (**C**) measurements. Each column is the mean of 4–6 individual plants, and bars represent standard error of the mean. Columns labeled with different letters, a, and b, represent significant differences between WT and transgenic lines at *p* < 0.05.

Soluble sugars, such as sucrose, glucose, and fructose, contribute to osmotic adjustment under drought conditions [57]. Concentrations of sucrose, glucose, and fructose were also measured in response to drought stress. Under control conditions, the concentrations of glucose and fructose were essentially similar in all genotypes (Figure 10). The content of sucrose in the transgenic seedlings was typically lower than that in the WT seedlings (Figure 10). However, both glucose and fructose contents increased in the transgenic plants following drought stress and were higher than those in the wild-type plants (Figure 10B,C). In contrast to hexose, the sucrose content markedly decreased in drought-stressed plants of WT and transgenic lines (Figure 10A).

## 3. Discussion

In this study, we confirmed the novel role of *K. candel* KcTrxf in the plant response to osmotic and drought stress. Phylogenetic analysis indicated that KcTrxf, with the canonical active site-WCGPC motif, is most homologous to Trxf orthologs (Figure 2). The KcTrxf protein was shown to localize to the chloroplast (Figure 3). Histochemical analysis of GUS expression showed that the *KcTrxf* promoter drives the expression of reporter genes in guard cells (Figure 4). Similarly, the promoter of pea thioredoxin f was shown to be active in the guard cells of transgenic Arabidopsis [37]. Enzymatic activity characterization revealed that KcTrxf recombinant protein possesses a disulfide reductase function, whereby it reduces the intermolecular disulfide bonds (Figure 5) [52,53,54].

Transcription analyses indicated that *KcTrxf* was upregulated by mannitol in *K. candel* leaves (Figure 1), suggesting that KcTrxf may contribute to osmotic tolerance. *KcTrxf* was overexpressed in tobacco to investigate its role in osmotic and drought tolerance. Our data from seedlings grown on MS medium and rooted plants acclimated to nursery soil showed that the tobacco plants overexpressing *KcTrxf* had enhanced osmotic and drought tolerance in terms of phenotypic screening. *KcTrxf*-transgenic plants exhibited greater root lengths and fresh weights under conditions of osmotic stress (Figure 6). Moreover, the *KcTrxf*-transgenic plants displayed less symptoms of wilting as compared to the wild-type plants under drought (Figure 7). These results show that KcTrxf enhances plant tolerance to osmotic stress and drought stress. Physiological data showed that enhanced tolerance of *KcTrxf*-transgenic plants mainly resulted from the increased ability to carry out stomatal control, osmotic adjustment, and ROS regulation under stress conditions.

The *KcTrxf* transgenic lines exhibited typically higher WRC than the wild-type plants during the period of air exposure (Figure 7). The lower water loss in transgenic lines resulted from the decreased stomatal aperture (Figure 7). As a result, the *KcTrxf*-transgenic plants were able to control the water content; consequently, leaf wilting was alleviated under drought conditions (Figure 7). NMT flux showed that *KcTrxf*-transgenic plants retained high K^+^ efflux in guard cells under drought conditions (Figure 8). Therefore, the increased stomatal sensitivity to drought presumably resulted from KcTrxf-enhanced efflux of K^+^, although further investigation is needed to determine the effects of the KcTrxf-mediated signaling pathway on K^+^ flux across the plasma membrane.

It is notable that KcTrxf increased ABA sensitivity in tobacco plants in terms of the stomatal response to the stress phytohormone (Figure 9). Drought generally increases the ABA level, which, in turn, activates the ABA signaling pathway in stress responses [31,32,56]. It has been shown that ABA activates K^+^ efflux channels in guard cells [31,36]. Thus, the increased K^+^ efflux in *KcTrxf*-transgenic plants might be associated with drought-induced ABA.

Under conditions of drought stress, *KcTrxf*-transgenic plants displayed a significantly lower MDA content than WT and VC plants (Figure 7). This indicates that *KcTrxf* overexpression helps plants to alleviate drought-induced oxidative damage, since MDA is a marker of lipid peroxidation [12,13]. The oxidative injury to WT plants was mainly the result of high H_2_O_2_ levels in transgene plants under drought conditions (Figure 7). Similarly, drought resulted in an increase in H_2_O_2_ accumulation and lipid peroxidation [19,20,21]. The accumulation of ROS in water-stressed plants impairs the function of biochemical processes, damages organelles, and ultimately results in cell death [58]. The less-affected water status in *KcTrxf*-transgenic plants could limit H_2_O_2_ production, as water shortage resulted in oxidative burst under drought stress conditions [58]. Moreover, KcTrxf might directly participate in the control of ROS under drought conditions. KcTrxf contains a redox-active dithiol in its active site (Figure 2), allowing it to serve as a redox regulator in prokaryotic and eukaryotic organisms [15,52,53,54]. Identification of putative target proteins by proteomics revealed that thioredoxin f1 interacts with other antioxidative defense systems such as glutaredoxins (Grx) in the chloroplasts [59,60,61]. In this study, drought treatment caused a decline in GSH, but it increased the GSSG concentration in WT plants, indicating that GSH contributed to ROS scavenging under conditions of drought stress [17,18,19]. Compared with WT, GSH was less affected in drought-stressed plants, and the drought increase in GSSG/GSH was less pronounced in *KcTrxf*-transgenic lines (Table 1). This indicates that transgenic plants are more efficient at regenerating GSH from GSSG by GR under drought stress conditions [17,19]. We previously showed that *KcTrxf*-transgenic plants have increased GR activity in the chloroplast AsA–GSH cycle, which enables plants to maintain GSH levels under conditions of NaCl stress [13]. Therefore, antioxidative enzymes and antioxidants such as glutathione in the AsA–GSH cycle enable transgenic plants to retain ROS homeostasis under drought conditions [17,19].

Soluble sugars play an important role in plant adaptation to water stress conditions [62]. Our data show that both glucose and fructose markedly increased in the *KcTrxf*-transgenic plants under drought stress conditions (Figure 10). This indicates that transgene plants could modify the osmolytes, allowing them to do osmotic adjustments to deal with drought stress [2,24]. The increased hexose concentration might result from starch degradation. Trx f1 has been shown to be involved in the regulation of starch degradation in *Arabidopsis thaliana* [47,48,49]. Thioredoxin-regulated β-amylase (BAM1) triggers diurnal starch degradation in guard cells and in mesophyll cells under osmotic stress conditions [48]. We noticed that the *KcTrxf*-transgenic plants displayed low sucrose levels under control and drought conditions (Figure 10). This suggests that KcTrxf affected the biosynthesis, storage, and mobilization of sucrose, since type-f thioredoxins have various functions in the mediation of carbon metabolism [15,45,46,47,48,49]. Glucose and fructose were found to increase f- and m-type *Trx* mRNA levels in *Pisum sativum*, while sucrose did not promote the expression of *Trxs* or even led to a decrease in the *Trx f* gene [63]. Therefore, we suggest that the high hexose content relative to sucrose might be favorable for sustaining the expression of *Trx f* and *m* genes in transgenic plants over a long period of time.

## 4. Materials and Methods

### 4.1. Plant Materials and Treatments

Propagules of *Kandelia candel* (L.) Druce were collected from Dongzhai harbor in Hainan province in China (latitude 19°51’N and longitude 110°24°E). The collected propagules were similar in size and were planted in individual 5-L pots containing sand in a greenhouse at Beijing Forestry University, China [9]. When the 4th pair of leaves came out, the seedlings were exposed to mannitol (0, 200 mM, 500 mM, and 1.0 M) in 500 mL of Hoagland nutrient solution for 8 h or 3 days. Then, the upper second leaves were immediately frozen in liquid N_2_ and stored at a freezer (−80 °C) for real-time quantitative polymerase chain reaction (RT-qPCR) analysis. Three biological replicates were used for RT-qPCR.

### 4.2. Full-Length KcTrxf Gene Cloning and Sequence Analysis

Total RNA was extracted from *Kandelia candel* leaves through a modified hot borate method [12]. RNA (1 μg) was used as a template to synthesize first-strand cDNA with oligo (dT) primer (Promega, Madison, Wisconsin, USA) and M-MLV Reverse Transcriptase (Promega, Madison, Wisconsin, USA). The full-length cDNA sequence of *KcTrxf* was amplified with specific forward (5′–TCAGCTTGATCTAGCAATCT–3′) and reverse primers (5′–TCAGCTTGATCTAGCAATCT–3′) [13]. The gel-purified PCR products were then ligated to the pMD18-T (Takara, Kusatsu, Japan) vector for DNA sequencing.

Amino acid sequences of Trxfs from different plant species were compared with ClustalW2 (http://www.ebi.ac.uk/Tools/msa/clustalw2/) (EMBL-EBI, Hinxton, Cambridgeshire, UK). The phylogenetic tree of Trxs was constructed by the neighbor-joining method with 1000 bootstrap replicates using MEGA 6.0 software (http://www.megasoftware.net/index.php) (Center for Evolutionary Medicine and Informatics, Tempe, AZ, USA).

### 4.3. Subcellular Localization Analysis

Full-length cDNA was obtained by PCR using the primers *Trxf*-forward 5′-GGAATT CATGGCTGATTCAATTCTCT-3′ and *Trxf*-reverse 5′-GGGGTACCCCGCTTTTCTAGCAATCTCA ATG-3′ [13]. The sequence was designed to contain restriction sites (an *Eco*RI site at the 5′ end and a *Kpn*I site at the 3′ end) and to eliminate the termination codon. Then, PCR products were digested by *EcoRI* and *KpnI* and introduced into pEZS-NL, which does not express GFP well without adding a coding sequence to the 5′ end of the open reading frame (https://deepgreen.dpb.carnegiescience.edu/; https://deepgreen.dpb.carnegiescience.edu/cell.imaging.site/html/vectors.html) (Carnegie Institution for Science, Washington, DC, USA). Arabidopsis mesophyll protoplast isolation and polyethylene glycol-mediated transformation were performed essentially in accordance with Yoo et al. (2007) [64]. Confocal images were obtained with a confocal laser scanning microscope (Leica Microsystems GmbH, Wetzlar, Germany) after 16 to 20 h of incubation. The intensity of fluorescence was examined at 510–535 and 650–750 nm for GFP and chlorophyll, respectively. The confocal parameters were set as described in previous studies: the excitation wavelength was 488 nm, and emission wavelength was 610–700 nm [65].

### 4.4. Construction of KcTrxf-pro::GUS and Transformation to Arabidopsis

The promoter region of *KcTrxf* (*KcTrxf*-pro) was isolated using the primers listed in Supplementary Materials Table S1 by hiTAIR-PCR, in accordance with the protocol described by Liu and Chen (2007) [66]. *KcTrxf*-pro was transferred into the pCambia1301 vector (forward 5′–GGAATTCCGGTCGTGGGTCCTCCTCCT–3′, reverse 5′–ACTGCCATGGTTTTGCCACTTGGGAA GAAAG–3′). The promoter region of *KcTrxf* was merged with the *GUS* gene and introduced into *Agrobacterium tumefacients* 3101 with a freeze–thaw method [67]. The *KcTrxf*-pro::GUS construct was transformed to Arabidopsis plants by the floral-dip method [68]. Arabidopsis carrying pCambia1301 was used as a negative control. Seedlings were incubated at 37 °C for 6 h with 1 mL of GUS substrate solution (0.5 mg ml^−1^ X-Gluc, 0.5 M sodium phosphate buffer pH 7.0, 1 mM potassium ferrocyanide, 1 mM ferricyanide, and 2% Triton-100). Green tissues were incubated in 70% ethanol for 24 h to remove chlorophyll. Samples were placed in 50% glycerol and examined under a dissecting microscope.

### 4.5. KcTrxf Expression and Purification of Recombinant Protein

The cDNA sequences encoding the KcTrxf protein were amplified by PCR using primers with restriction sites (forward 5′-GGAATTCATGGCTTGATTCAATTCTCT-3′, reverse 5′-CCGCTCGAGTCAGCTTGATCTAGCAATCT-3′) and cloned into the pET28a expression vector *Eco*RI/*Xho*I sites. The recombinant mutant KcTrxf (amino acids 63–194) without the putative transit peptides was cloned into the *Eco*RI/*Xho*I sites of the pET28a expression vector. The resulting constructions were introduced into the *Escherrichia coli* BL21, and recombinant protein expression was induced by the addition of 0.1 mM isopropylthythio-β-galactoside at 28 °C overnight. The recombinant proteins were purified through Ni^+^ affinity chromatography. Thereafter, the purified proteins were analyzed by SDS-PAGE.

### 4.6. Trxf Activity Assay

The purified KcTrxf recombinant protein was used to examine activity of Trx. Trx activity was assessed using the insulin reduction assays according to a modified protocol from Holmgren (1979) [55]. The incubation mixture contained 0, 3, or 5 µM of His_6_-tagged KcTrxf protein in 100 mM of potassium phosphate buffer at pH 7.0 with 2 mM of ethylenediaminetetraacetic acid (EDTA), 0.13 mM of bovine insulin, and 0.25 mM of dithiothreitol as a reductant. The turbidity of the reduced insulin chains was recorded at 650 nm for 30 min. The Trxf activity measurements were repeated three times.

### 4.7. KcTrxf Transformation in Tobacco

Overexpression of *KcTrxf* in tobacco was performed as previously described [13]. In brief, the open reading frame (ORF) of *KcTrxf* was cloned and introduced into a donor vector-Dtop with the gateway methods. The cDNA in Dtop-Trx was introduced into PK7 to form the expression vector PK7-TRX. The construct was transformed to *Agrobacterium tumefacients* LBA 4404 with a freeze–thaw method [67]. The *A. tumefacients* strain was transferred to *Nicotiana tabacum L.* by the leaf-disc method [13,67]. The *A. tumefacients*-infected leaves were placed on MS without antibiotics for 2–3 days and transferred to MS supplemented with 50 mgL^−1^ of kanamyein (Kan) and 100 mgL^−1^ of carbenicillin (Carb). After 3–4 weeks, individual kanamycin-resistant shoots were selected and were cultured in MS medium without growth regulators or antibiotics. More than 20 independently transformed plants were screened for expression of *KcTrxf* [13], three of which with higher expression levels (L9, L10, and L21) were selected for further study. Then, plants were moved to soil to obtain seeds for further study. The soil-cultured plants were transferred to growth chambers with a 16 h photoperiod (150 µmol m^−2^·s^−1^ irradiation, 21 °C, 80% relative humidity).

### 4.8. Real-Time Quantitative PCR

The expression of *KcTrxf* in *Kandelia candel* and transgenic tobacco was analyzed using RT-qPCR with specific primers: forward 5′-TGGTTGCCATTGAGATTGC-3′ and reverse 5′-CCCAAATCGGAAGATGATA-3′. Total RNA was extracted from *Kandelia candel* from the second leaves of at least three different plants by a modified hot borate method [12], whereas the total RNA of *Nicotiana tabacum* was extracted from the second leaves of at least three independent plants using the Trizol method [13]. The first strand was synthesized from 2 µg of total RNA using the RNase M-LV and oligo (d_T_)12–15 primer at 42 °C for 1 h. The real-time PCR conditions were 10 min at 95 °C; 35 cycles of 95 °C for 30 s, 55 °C for 30 s, and 72 °C for 30 s; followed by 10 min at 72 °C. The endogenous housekeeping genes *Tublin* and *EF1α* were used for *Kandelia candel* and *Nicotiana tabacum,* respectively. RT-qPCR was performed using the following primers: (i) Tubulin—forward 5′-TGCCCAAGGATGTGAACG-3′, reverse 5′-CCATACCCTCACCCACAT-3′; (ii) *EF1α*—forward 5′-CTGTGAGGGACATGCGTCAAA-3′, reverse 5′-GTAGTAGATCGCGAGTACCACCA-3′). The relative expression was quantified by MJ Opticon Montor software 3.1 (Bio-Rad Laboratories, Inc., Hercules, CA, USA). Three biological replicates were used for each round of RT-qPCR.

### 4.9. Phenotype Tests to Assess Osmotic and Drought Tolerance

Using genomic PCR, T2 generation of L9, L10, and L21 was checked for the presence of the *KcTrxf* gene. Seeds of wild-type (WT) and transgenic lines (L9, L10, and L21) were germinated on MS medium for 7 days and then subjected to 250 mM of mannitol for 21 days. The root lengths and fresh weights of whole seedlings were measured in WT and transgenic lines. Twelve to 20 individual plants were used for each treatment. Four-week-old rooted plants of WT and transgenic lines were transferred to nursery soil for 4 weeks of acclimation and then exposed to drought by withdrawing water for 2 weeks. The water-retaining capacity, stomatal aperture, malondialdehyde (MDA) content, H_2_O_2_ production, soluble sugar content in leaves, and K^+^ flux in guard cells were examined in control and stressed plants of WT and transgenic lines. All measurements were performed on the third or fourth fully expanded leaf.

### 4.10. Determination of MDA, H_2_O_2_, GSH, and GSSG

Oxidative damage to lipids was determined by measuring the content of MDA according to the method described by Jing et al. (2015) [12] and Deng et al. (2015) [65]. Leaf samples (0.1 g) were frozen in liquid nitrogen and homogenized in 2 mL of 0.1% (*w*/*v*) thiobarbituric acid (TBA). The homogenate was centrifuged at 10,000 rpm for 5 min. Then, 2 mL of 0.5% (*w*/*v*) TBA in 20% (*v*/*v*) trichloroacetic acid (TCA) was added to a 0.5-mL aliquot of the supernatant. Samples were heated at 95 °C for 30 min and then quickly cooled in an ice bath for 15 min. After 10 min of centrifugation at 10,000 rpm and 4 °C, the absorbance was measured at 450, 532, and 600 nm, respectively. The content of H_2_O_2_ was measured by monitoring the A_415_ of the titanium peroxide complex. Absorbance values were calibrated to a standard curve generated with known concentrations of H_2_O_2_. Leaf samples from four individual plants were used for each treatment.

The GSH and GSSG contents were determined as described by Griffith (1980) [69]. Briefly, leaf samples (0.1 g) were frozen in liquid nitrogen and homogenized in 1 mL of 2.5 M HClO_4_ for 10 min. Then, the homogenate was centrifuged at 10,000 rpm for 10 min and the pH was adjusted 6.3–6.7 by 1.25 M Na_2_CO_3_. The GSH content in 1 mL of mixture (100 µL supernatant, 100 µL of 6 mM 5, 5-dithiobis-2-nitrobenzoic acid (DTNB), 800 µL of 0.3 mM reduced nicotinamide adenine dinucleotide phosphate (NADPH), and glutathione reductase (GR, 1 U)) was measured by monitoring the absorbance at 412 nm. To determine the GSSG content, vinyl pyridine (2 µL) was introduced to the supernatant and incubated for 1 h. Then, the absorbance of the mixture was measured as described above. Absorbance values were calibrated to a standard curve generated with known concentrations of GSH.

### 4.11. Water-Retaining Capacity

Four-week-old rooted plants of WT and transgenic lines were transferred to nursery soil for 4 weeks of acclimation. Then, the upper mature leaves (the third to fifth leaves from the tip) were excised to measure the water-retaining capacity. Leaf samples were placed on a laboratory bench under a light intensity of 150–200 μmol m^−1^ s^−1^ at 25 °C and a relative humidity of 40%. Water loss from the leaf surface was measured during the period of 120 min air exposure [70]. Four individual plants were used for WT plants and each transgenic line.

### 4.12. Stomatal Aperture Measurement

The abaxial epidermis was carefully stripped from control and droughted plants of WT and transgenic lines. The stomatal apertures were measured under a microscope using the image processing software ImageJ (National Institutes of Health, Bethesda, MD, USA).

For ABA treatment, epidermal peels were stripped from WT and transgenic lines and floated in the opening solution (30 mM KCl and 10 mM 2-morpholinoethanesulfonic acid (MES)–KOH, pH 6.15) for 2.5 h under cool, white light. Then, ABA stock solution was added to the opening solution to reach a final concentration of 5 µM. The control was treated without the addition of ABA. After 2 h of ABA treatment, the stomatal apertures were measured as described above. One hundred stomata from four individual plants were used for WT and each transgenic line.

### 4.13. K^+^ Flux in Guard Cells

Tobacco leaves were sampled from control and droughted plants of WT and transgenic lines. The leaves were washed and rinsed with redistilled water. The abaxial epidermis was carefully stripped from the leaves, and epidermal peels were immediately incubated in measuring solution containing the following components (in mM): KCl (0.5), CaCl_2_ (0.1), MgCl_2_ (0.1), NaCl (0.1), and 2.5% sucrose (the pH of the solution was adjusted to 5.8). After immobilization on the bottom of the chamber, steady flux profiles of K^+^ in guard cells were recorded with the Noninvasive Micro-Test Technique (NMT-YG-100, Younger USA LLC, Amherst, MA, USA) with ASET 2.0 (Sciencewares, Falmouth, MA 02540, USA) and iFluxes 1.0 (Young-erUSA, LLC, Amherst, MA 01002, USA) software, which is capable of integrating and coordinating differential voltage signal collection, motion control, and image capture simultaneously. The K^+^ flux was measured by shifting the ion-selective microelectrode between two sites close to the guard cells at a frequency in the range of 0.3–0.5 Hz [71]. For WT and each transgenic line, 30–40 stomata from four individual plants were used for control and drought treatment.

### 4.14. Soluble Sugar Measurement

Glucose, fructose, and sucrose were measured by the enzymatic method [72]. After being ground in liquid nitrogen, samples (100 mg) were heated in triplicate at 80 °C for 20 min and centrifuged for 5 min at 14,000 rpm after successive addition of 500 μL of 80% (v/v) ethanol. Supernatants were combined, and the ethanol was removed by rotary evaporation. The residue was dissolved in 100 µL of water and used for soluble sugar determination. A glucose (HK) assay kit was used for the determination (Sigma GAHK-20, Sigma-Aldrich, St. Louis, MO, USA)). Then, 10 μL of 100 U mL^−1^ phosphoglucomutase and 10 μL of 100 μg L^−1^ invertase were added in succession to determine fructose and sucrose contents. For each treatment, 4–6 individual plants were used for WT and each transgenic line.

### 4.15. Data Analysis

All experimental data were analyzed using SPSS (version 19.0, IBM Corporation, Armonk, New York, USA) software. Statistical analyses were performed using one-way ANOVA. Unless otherwise stated, a *p*-value of less than 0.05 was considered statistically significant.

## 5. Conclusions

Mannitol treatment induced the expression of thioredoxin f, *KcTrxf*, in *K. candel*. Amino acid sequencing and phylogenetic analysis of the mangrove Trx classified it as an f-type Trx. Subcellular localization revealed that *K. candel* Trxf localized to chloroplasts. Enzymatic activity characterization revealed that the KcTrxf recombinant protein possessed Trx activity. *KcTrxf* overexpression contributed to osmotic and drought tolerance in tobacco. KcTrxf was shown to reduce the stomatal aperture by enhancing K^+^ efflux under drought conditions, which increased the water-retaining capacity. Notably, the ABA sensitivity was increased in *KcTrxf*-transgenic tobacco, enabling the plants to sense and transduce stress signals after the onset of drought, thus limiting water loss through transpiration during prolonged periods of water stress. *KcTrxf*-transgenic plants had limited drought-induced H_2_O_2_ in leaves, which could reduce lipid peroxidation and retain the membrane integrity. In addition, GSH contributed to ROS scavenging and transgenic plants are more efficient at regenerating GSH from GSSG under conditions of drought stress. More importantly, *KcTrxf*-transgenic plants had increased glucose and fructose contents under drought stress conditions, most likely resulting from Trxf-promoted starch degradation under water stress. We conclude that KcTrxf contributes to osmotic and drought tolerance by increasing WRC, by enhancing osmotic adjustment, and by maintaining ROS homeostasis in transgenic plants. Therefore, *KcTrxf* has great potential for use in genetic transformation of drought susceptible species, for example, fast-growing poplars, which are used for large-scale plantations and afforestation in the dry lands of northern China.

## Figures and Tables

**Table 1:** The contents of glutathione (GSH) and oxidized glutathione (GSSG) and the ratio of GSSG/GSH in leaves of wild-type tobacco and *KcTrxf*-transgenic seedlings under conditions of drought stress: Four-week-old rooted plants on the MS of WT and transgenic lines (L9, L10, and L21) were transferred to nursery soil for 4 weeks of acclimation and then exposed to drought treatment by withholding water for 2 weeks. Control and stressed plants were harvested from WT and transgenic lines to measure GSH and GSSG contents. Each value (±SD) is the mean value of four individual plants, and values labeled with different letters, a, b, and c, represent significant differences between WT and transgenic lines at *p* < 0.05.

	Lines	Control	Drought
GSH	WT	28.01 ± 0.16^a^	24.34 ± 0.71^a^
	Line 9	16.50 ± 0.38^c^	17.68 ± 0.17^b^
	Line 10	15.57 ± 0.65^c^	15.38 ± 0.72^b^
	Line 21	20.87 ± 0.38^b^	15.91 ± 0.24^b^
GSSG	WT	16.48 ± 0.73^b^	21.40 ± 0.92^a^
	Line 9	18.87 ± 0.44^ab^	21.97 ± 0.87^a^
	Line 10	18.42 ± 0.68^ab^	21.55 ± 0.11^a^
	Line 21	19.51 ± 0.77^a^	20.11 ± 0.71^a^
GSSG/GSH	WT	0.59 ± 0.02^c^	0.88 ± 0.02^b^
	Line 9	1.14 ± 0.03^a^	1.24 ± 0.04^a^
	Line 10	1.18 ± 0.05^a^	1.40 ± 0.06^a^
	Line 21	0.93 ± 0.04^b^	1.26 ± 0.05^a^

## Supplementary Materials

The following are available online at https://www.mdpi.com/1422-0067/21/9/3335/s1, Table S1: Primer sequences used for cloning *Kandelia candel Trxf* promoter.

## Abbreviations

ABA	abscisic acid
DTNB	5, 5-dithiobis-2-nitrobenzoic acid
EDTA	ethylenediaminetetraacetic acid
GR	glutathione reductase
GSH	reduced glutathione
GSSG	oxidized glutathione
H_2_O_2_	hydrogen peroxide
	
MDA	malondialdehyde
MDAR	monodehydroascorbate reductase
MS	Murashige–Skoog medium
MES	2-morpholinoethanesulfonic acid
NADPH	reduced nicotinamide adenine dinucleotide phosphate
NMT	noninvasive micro-test technique
NPTs	nonprotein thiols
ORF	open reading frame
RT-qPCR	real-time quantitative PCR
ROS	reactive oxygen species
SOD	superoxide dismutase
PM	plasma membrane
TRX	thioredoxin
TBA	thiobarbituric acid
TCA	trichloroacetic acid
WRC	water-retaining capacity
