# Supplementary material for: Kandelia candel Thioredoxin f Confers Osmotic Stress Tolerance in Transgenic Tobacco
Source: Int J Mol Sci. 2020 May 8;21(9):3335. doi: 10.3390/ijms21093335 (PMC7247566; doi:10.3390/ijms21093335)
Supplement: Supplementary file 1 [file ijms-21-03335-s001.pdf]

Supplementary Table S1. Primer sequences used for cloning *Kandelia candel Trxf* promoter

**Primers**	**Sequences (5′ to 3′)**
LAD1-1	ACGATGGACTCCAGVNVNNNGGAA
LAD1-2	ACGATGGACTCCAGBNBNNNGGAA
LAD1-3	ACGATGGACTCCAGVVNVNNNGGAA
LAD1-4	ACGATGGACTCCAGBDNBNNNGGAA
AC1	ACGATGGACTCCAG
SP1	ACCCACCGTGTCCAAGCTCGACT
SP2	ACGATGGACTCCAGTCTCTTAGCATTGAGGTCGGCAGATT
SP3	ATGGAAGAGGATGCGGACGAGT
